# Clinical Relevance of Bone Density Values from CT Related to Dental Implant Stability: A Retrospective Study

**DOI:** 10.1155/2018/6758245

**Published:** 2018-05-31

**Authors:** Vincenzo Bruno, Cesare Berti, Carlo Barausse, Mauro Badino, Roberta Gasparro, Daniela Rita Ippolito, Pietro Felice

**Affiliations:** ^1^Department of Biomedical and Specialty Surgical Sciences, Unit of Prosthesis, University of Ferrara, Via Borsari 46, 44121 Ferrara, Italy; ^2^Department of Biomedical and Neuromotor Sciences, Unit of Periodontology and Implantology, University of Bologna, Via San Vitale 59, 40125 Bologna, Italy; ^3^Private Practice, Vicolo Carceri 10, 10064 Pinerolo, Torino, Italy; ^4^Department of Neuroscience, Reproductive Sciences and Odontostomatology, Unit of Oral Surgery and Implantology, University of Naples, Via Pansini 5, Edificio 14, 80131 Naples, Italy; ^5^Department of Biomedical and Neuromotor Sciences, Unit of Orthodontics, University of Bologna, Via San Vitale 59, 40125 Bologna, Italy

## Abstract

**Purpose:**

The majority of the techniques used to assess the primary implant stability are subjective and empirical and can be used during or after the surgery. The aim of this study is to evaluate the bone density prior to surgery, in order to give recommendations to the clinician about the best surgical technique and the type of implant which is needed.

**Materials and Methods:**

A surgeon operated on 75 patients for 269 implants over the period 2010–2014. He required a CT to plan the surgery and he documented the type, the diameters, and the lengths of the implants, the insertion torque, and the ISQ values. At a later stage another clinician measured bone density and cortical thickness. We endeavoured to get the most accurate superimposition between the implants placed by the surgeon and those placed by the clinician.

**Results:**

In maxilla ISQ showed a significant positive correlation with HU values detected for coronal-buccal (*r* = 0.302; *p* = 0.020) and middle-lingual (*r* = 0.295; *p* = 0.023). Torque showed a positive correlation with cortical bone thickness at the middle of the ridge (*ρ* = 0.196; *p* = 0.032).

**Conclusion:**

It is important to take into consideration the Hounsfield Units and the cortical thickness as predictive parameters during the preoperative assessment, with regard to the choice of the implant type as well as the surgical technique.

## 1. Introduction

Osseointegration underlies contemporary implantology and it occurs in a primary and secondary level [1]. The primary implant stability can be defined as the “biometric stability immediately after implant insertion” [2], a mechanical phenomenon that is related to the local bone quality and quantity, to the implant geometry (i.e., length, diameter, and type), and to the placement technique used (i.e., relation between drill size and implant size, whether a pretapped or self-tapped implant is used). The primary implant stability has always represented one of the essential prerequisites for performing and maintaining osseointegration [3], for it prevents micromovement and the formation of fibrous scar tissue at the time of implant loading. Unfortunately, the majority of techniques for testing implant stability are widely empirical and subjective; moreover they evaluate the bone quality during or after (RFA and Periotest) the implant surgery [4]. All these methods are useful in evaluating the osseointegration, but no objective information on bone quality has been given prior to the preparation of the osteotomy [5, 6]. Moreover, according to Degidi et al. [7]: “the primary implant stability prediction is not good enough to prevent mistakes when using for example an immediate loading technique”. A method, which is proven to be objective and valid to assess the bone density prior to surgery, is to utilize the HU value measured on CT images [8–10]. The aim of this prospective study is to evaluate the bone density prior to surgery, in order to give recommendations to the clinician about the best surgical technique and the type of implant which is needed. Thanks to this information, the clinician could be able to obtain the best primary implant stability, essential to obtain a long-term success, even in those cases where the bone is not particularly dense; this includes pertinent information regarding the diameter, the length, and the type of implant. In this study, the correlation between the Hounsfield Units (5 values around the implant) from the Computerized Tomography, the width of the cortical bone (3 values around the implant neck), and the final insertion torque and the resonance frequency (ISQ) were all evaluated.

## 2. Materials and Methods

### 2.1. Enrollment of Patients

An experienced implantologist had consecutively enrolled 75 patients for 269 implants over the period 2010–2014. He required a conventional multislice computed tomography (CT) to assess bone quantity and to ensure that there was sufficient bone to perform surgery without the need of a bone augmentation procedure, prior to implant placement. The reason we used CT was that at the time we were unable to use cone beam computed tomography. All patients gave written informed consent and the guidelines of the Declaration of Helsinki were observed.

### 2.2. Baseline Measurement

**Figure 1:**
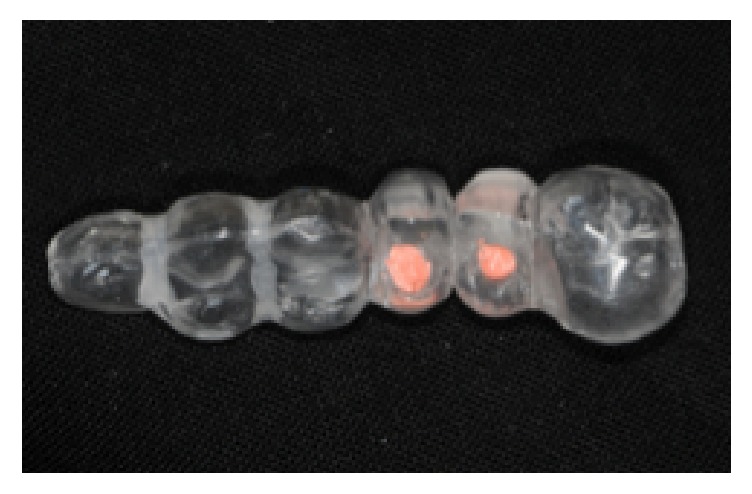
A radiological template with gutta-percha marks.

**Figure 2:**
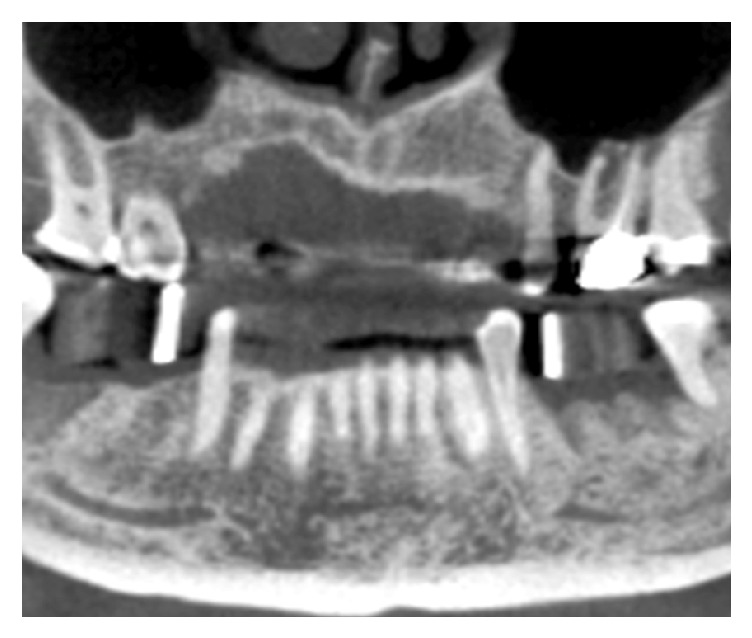
A panoramic view from CT with the radiological template in situ.

**Figure 3:**
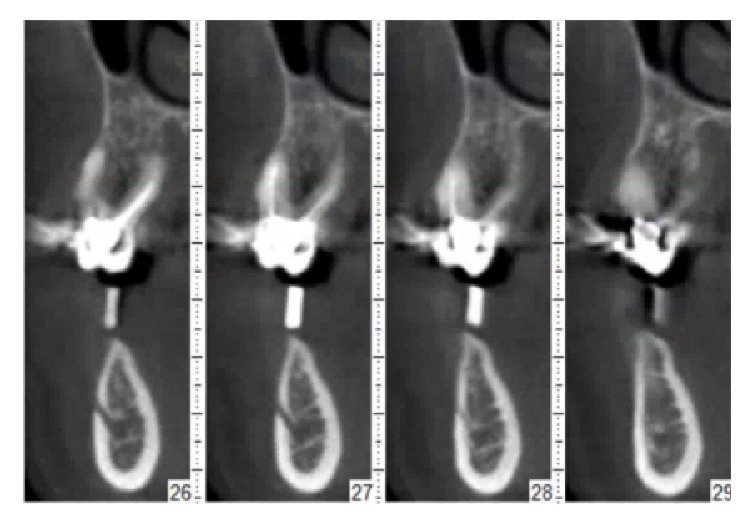
Some slices of the CT.

**Figure 4:**
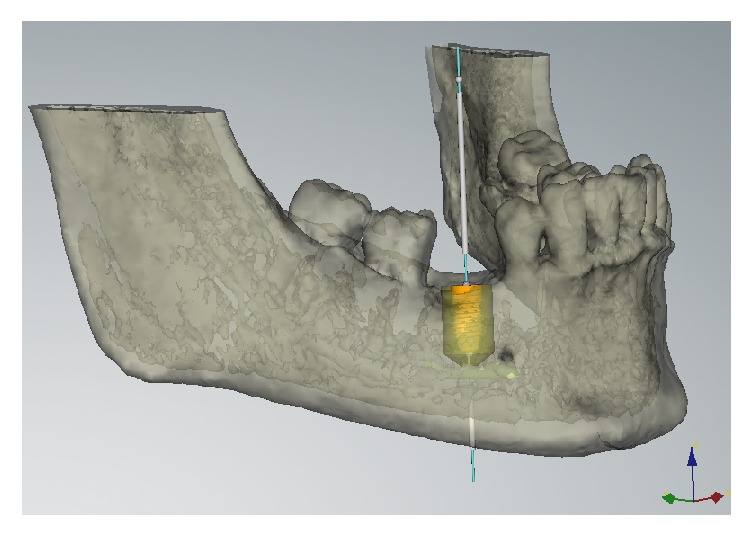
The implant planned by the surgeon in the NobelClinician software selected from the implant.

Alginate impressions were taken and diagnostic casts were fabricated (Vel-Mix Die Stone, Kerr Corporation, Washington, DC). A transparent template by using a clear acrylic resin (ProBase Cold; IvoclarVivadent AG, Schaan, Liechtenstein), based on the wax up, was constructed and gutta-percha marks were inserted along the axis of the teeth to be replaced (Figure 1). A CT was obtained with the template placed in situ (Figures 2 and 3). All CTs were performed using identical settings which were applied for all patients: 120 kV, 90 mAs, 0.5 mm slice thickness, and 0.3 mm slice increment. Data was stored in Dicom format. These Dicom files were loaded in a planning software (NobelClinician, Nobel Biocare AB, Goteborg, Sweden), which included 3D reconstruction and drawing of the reference curve. This procedure allowed for localization of the osteotomy sites related to the gutta-percha marks and, therefore, the corresponding cross-sectional CT slices. The surgeon planned the implant(s) (Nobel Replace Select Tapered, Nobel Active, Nobel Replace Select Straight, Nobel Replace Groovy, Nobel Speedy, Branemark Groovy), including the length and diameter, and selected an implant model, from the NobelClinician implant library, and was/were finally placed at the corresponding site(s). The surgeon, at that time, did not take any other measurements (Figure 4).

### 2.3. Surgical Procedures

The template was modified with holes in the implant positions to perform a precise surgery. Preoperative antibiotics were given orally 1 day prior to surgery and were continued for another 5 days, every 12 hours, prescribing amoxicillin, 1 g. The surgery was performed using a full thickness mucoperiosteal flaps which were raised under local anaesthesia. A 2 mm diameter twist drill was used, under profuse isotonic saline irrigation, to prepare the initial full depth channel at the implant site. The sequence of tapered drill of the length and the diameter chosen was then used to shape the osteotomy site, always using profuse irrigation. Finally, implants were inserted without the use of the irrigation.

### 2.4. Data Collection

The surgeon proceeded with the surgery using the modified template, documenting the diameters and the lengths of the implants, and the insertion torque (20, 35, 45, and more than 45 Ncm) until the implant reached its final position. Finally, the Resonance Frequency Analysis (RFA) was recorded using the wireless device (Ostell ISQ Instrument, Integration diagnostics AB, Sävedalen, Sweden), measuring the ISQ values in four different directions consisting of the mesial, distal, lingual, and buccal which calculated the mean ISQMean, for 109 implants.

### 2.5. Measurements of Bone Density and Cortical Thickness

**Figure 5:**
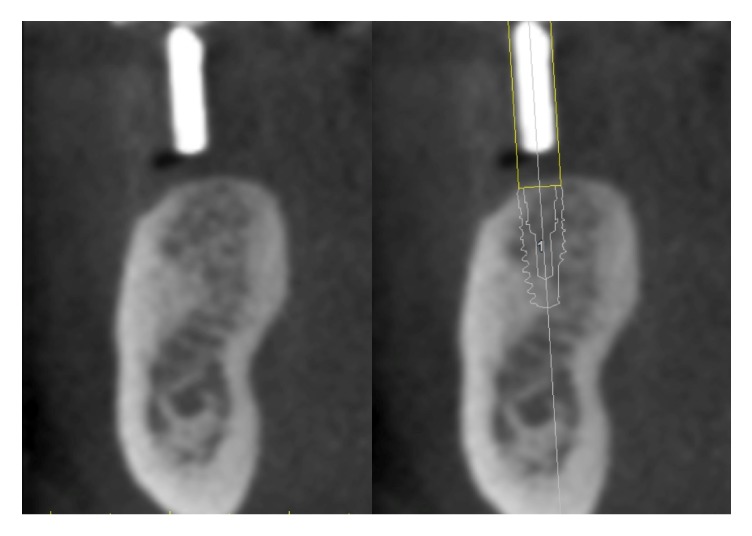
Superimposition between the gutta-percha mark and the implant placed by the clinician.

**Figure 6:**
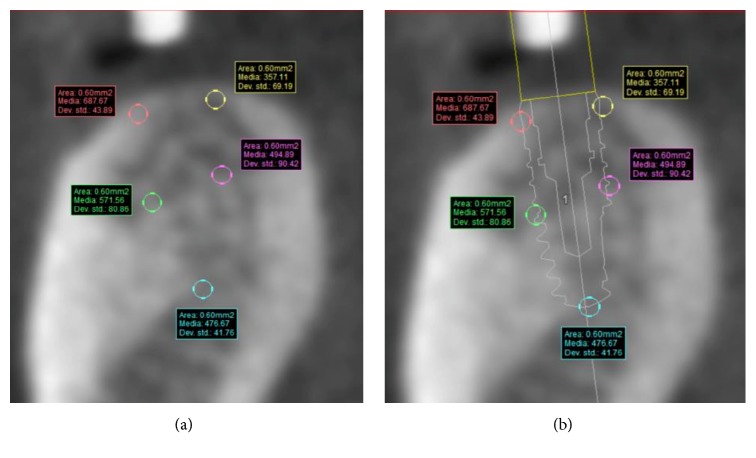
(a) The five points where to measure the Hounsfield Units and (b) in relation to implant.

**Figure 7:**
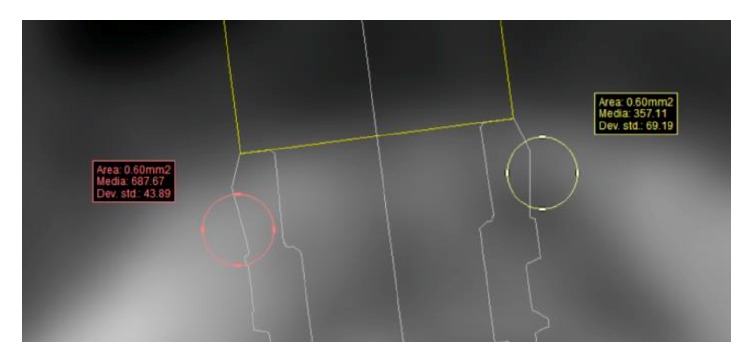
HU values detected as the arithmetical means of an area measuring 60 mm^2^.

**Figure 8:**
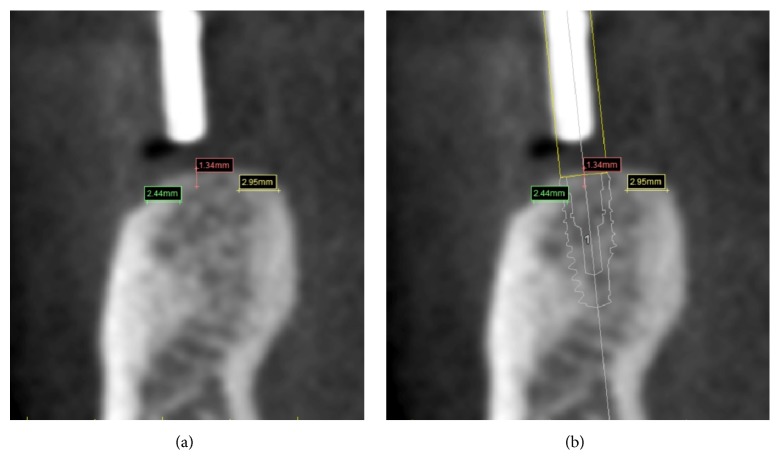
The three lines designed to measure the Cortical Thickness and (b) in relation to implant.

The measurements of bone density and cortical thickness were obtained by another clinician to avoid any bias, using the SIMPLANT® software. This clinician was not aware of other parameters except for the diameter, the lengths, the type of implants used, and their position. The measurements were carried out at a later stage than the surgery. The clinician simulated the implants' position by using, as reference points, the same gutta-percha marks—the same which the surgeon had used in order to plan the surgery and find the best implant site for each patient; that way, we endeavoured to get the most accurate superimposition between the implants placed by the surgeon and those placed by the clinician, so as to study bone density and cortical thickness (Figure 5). The assessment was made at 5 points on every slice: coronal-buccal (HU1), middle-buccal (HU2), middle at the apex (HU3), middle-lingual (HU4), and coronal-lingual (HU5) (Figures 6(a) and 6(b)). The Hounsfield Units (HU) values were recorded separately, and they were calculated as the arithmetical means of an area measuring 60 mm^2^ for each of the five spots around the implant which we examined (Figure 7). Additionally, the cortical thickness was calculated at sites of the implant–bone contact: lingual (C1) and buccal (C2), including the middle of the ridge (C3). The measurements of cortical thickness were carried out as material measures around the implant neck (Figures 8(a) and 8(b)). The clinician selected the implants from the SIMPLANT® implant library, using the type of implants and the same diameter and length that the surgeon chooses for the surgery.

### 2.6. Statistical Analysis

A sample size calculation was conducted. The primary outcome on which the calculation was based was the difference in mean torque among the different implant types. Given *α* = 0.05, *β* = 0.80, a medium effect size (*f* = 0.25), 6 groups, and an additional 15% samples compared to the corresponding parametric test, the minimum sample required was determined to be 249. All data analyses were carried out according to a pre-established analysis plan. The implant was the statistical unit of the analyses. A dentist with expertise in statistics analyzed the data without knowing the group allocation. Data were summarized using frequencies (for nominal-level variables), means, and standard deviations (for continuous data). Independent* t*-tests and Mann–Whitney tests were used to examine the differences, respectively, in ISQ and torque between genders and implant location (anterior or posterior). One-way ANOVAs and Kruskal Wallis tests explored the differences in ISQ and torque among subjects with different smoking habits. Relationships between ISQ and continuous variables (age, bone density according to the Hounsfield scale, cortical bone thickness, implant length, and implant diameter) were assessed by using the Pearson Product-moment correlation. A Spearman's Rank Order correlation was run to determine the relationship between torque and continuous variables. Differences in ISQ among different implant types were investigated through a One-way ANOVA. To control for the continuous variables which had shown a significant correlation with ISQ an ANCOVA was used; a two-way ANOVA was conducted to examine the effect on ISQ of nominal variables which had shown significant differences in ISQ together with implant type. A Kruskal Wallis test was used to investigate the differences in torque among different implant types. Pairwise comparisons were performed by using the Mann–Whitney *U* test with Bonferroni correction (maxilla: adjusted *α* level = 0.003; mandible: adjusted *α* level = 0.005). Ordinal regressions were used to analyze the effect of the interaction between type of implant and the variables which had shown a significant correlation with torque. All statistical analyses were conducted using the Statistical Package for Social Sciences Software (IBM Corp. Released 2012. IBM SPSS Statistics for Windows, Version 21.0. Armonk, NY: IBM Corp). *p* < 0.05 was set as the level for statistical significance.

The data that support the findings of this study are available on request from the corresponding author. The data are not publicly available due to privacy reasons.

## 3. Results

Seventy-five patients were enrolled in the study. The main baseline patient characteristics are presented in Table 1. No significant differences were found in ISQ according to patient characteristics (sex, age, and smoking habits) nor in maxilla nor in mandible (*p* > 0.05). Torque in mandible showed a significant negative correlation with age (*ρ* = −0.236; *p* = 0.009). Concerning the bone characteristics (implant location, bone density according to the Hounsfield scale, and cortical bone thickness), in maxilla ISQ showed a significant positive correlation with HU values detected for coronal-buccal (*r* = 0.302; *p* = 0.020) and middle-lingual (*r* = 0.295; *p* = 0.023). In mandible a significant difference was found between anterior and posterior implant location both in ISQ (anterior: 66.04; posterior: 72.22; mean difference: −6.182; 95% CI of the difference: −10.033 to −2.331; *p* = 0.002) and in torque (anterior: 1.81 ± 1.05; posterior: 2.47 ± 0.98; *p* = 0.006). Moreover torque showed a positive correlation with cortical bone thickness at the middle of the ridge (*ρ* = 0.196; *p* = 0.032). With respect to implant characteristics, a statistically significant correlation was found between ISQ and implant length in maxilla (*r* = 0.316; *p* = 0.015) and between torque and implant length both in maxilla (*ρ* = 0.216; *p* = 0.008) and in mandible (*ρ* = −0.318; *p* < 0.001). A significant correlation between torque and implant diameter was found both in maxilla (*ρ* = 0.172; *p* = 0.036) and in mandible (*ρ* = 0.370; *p* < 0.001) whereas no significant correlations existed between ISQ and implant diameter (*p* > 0.05). No significant differences were found in ISQ among the different implant types (Table 2), even after controlling for the variables that had shown a significant correlation with ISQ (*p* > 0.05; Tables 3 and 4). No statistically significant interactions between the effects on torque of implant type and each variable that had shown a significant correlation with torque were found (*p* > 0.05). In maxilla Nobel Active implants showed a significantly higher torque than Nobel Replace Select Straight implants, Nobel Replace Groovy implants, and Nobel Speedy implants; in mandible Nobel Replace Select Tapered implants had a significantly higher torque than Replace Select Straight implants (Table 5).

## 4. Discussion

Several published methods are suggested for the assessment of bone quality, but many of these have shown a deficiency in the objectivity [6, 11, 12], because they are dependent upon the practitioner and/or can only be used during or after surgery [4]. Percussion and manual testing assess the implant stability by judging the presence of any mobility by a gentle application of a soft rotational force to the implant and abutment complex by the use of an appropriate screwdriver. They are widely practised clinical techniques, but there is little evidence in the literature supporting these concepts [12]. Radiographic examination is the most commonly used technique in clinical practice [13]. The implant is monitored at 6 and 12 months and every year thereafter in order to identify any marginal bone loss and perifixtural radiolucencies. Unfortunately, radiographs have a poor diagnostic ability for detection of perifixtural radiolucency due to their limited discriminatory acuity [14] and they show a two-dimensional image, while the implant/bone interface is a three-dimensional area; moreover, it is difficult to use a standard technique to ensure good reproducibility [15]. The insertion torque records the torque required to place the implant, it provides important information about the local bone quality and maybe about primary stability; indeed several authors have reported that the insertion torque measurements can be used to determine primary stability [16–20]. Removal torque is a technique that involves measuring the peak torque necessary to shear the interface between the implant surface and the surrounding bone, with a manual torque gauge; this test has been criticized as being destructive method and it is mostly used only in experiments [21]. Another method can be used after the implant placement is the Periotest® (Siemens AG, Bensheim, Germany) [22], which consists of percussing implant surface with a handheld probe containing an electromagnetically driven metal pellet; the mobility is assessed by the measurement of the contact time between the metal hammer and the surface under test. The sensitivity of Periotest to clinical variables such as striking position and hand piece angulation limited the application of the instrument as a definitive clinical diagnostic tool, to be used; the values of this method are also influenced by the implant and abutment lengths [5]. With the advent of Resonance Frequency Analysis, there is an objective method for stability testing [23]: it analyses the resonance frequency of a transducer attached to an implant fixture or abutment [24]. The most recent version of Resonance Frequency Analysis is wireless, where a metal rod is connected to the implant by means of a screw connection (Osstell ISQ instrument). This is a consistent and noninvasive technique to establish clinically relevant information about the state of the implant–bone interface at any stage of the treatment or at follow-up examinations [25]. In this study we evaluated the correlations between diameter and implants lengths, HU values, and cortical thickness with ISQ and the insertion torque (both objective parameters to assess the primary implant stability [26, 27]) in order to evaluate the bone density prior to surgery and give recommendations to the surgeon about the best surgical technique and about the type of implant; this was suggested by Salimov et al. [28], who explored the efficacy of bone density values by evaluating its correlation with the implant stability parameters, including insertion torque value and the Resonance Frequency Analysis, finding a correlation, however with the limit of a clinical study on just 17 patients. We carried out the present study using preexisting CTs because, at the time of treating these patients (2010–2014), we were unable to use CBCT. Moreover, being not able at the time to acquire any CTs in our private practice, patients were simply referred, so different CT machines were used. Anyway, there is a significant correlation between primary implant stability and gray density values detected not only by cone beam (CBCT), but also by conventional multislice computed tomography (CT). In any case, CBCT is nowadays preferable because of lower radiation dose and costs, as stated by Arisan et al. [29]; as a consequence we have planned to publish our results with CBCT, once we reach a suitable number of patients with adequate follow-ups. In our study we found that the cortical bone thickness in the middle of the ridge showed a positive correlation with torque, and it could be considered in an agreement with the conclusions of other studies [30, 31] on the importance of cortical thickness as a predictive factor of primary implant stability. Additionally, it could be interesting to highlight that crestal cortical bone thickness depends on the region of the jawbone. The thickness shows the highest values in posterior mandible and the lower in posterior maxilla: that leads us to pay close attention to implant placement in the posterior maxilla region in order to obtain a good primary implant stability [32]. The present study also found a significant positive correlation between ISQ and the HU values detected for coronal-buccal and middle-lingual in maxilla, according to Turkyilmaz et al. [9], which found statistically significant correlations between ISQ and bone density, expressed in HU values. Moreover, we found that the posterior implant location in mandible showed higher values of both ISQ and torque than the anterior implant location in the same jaw. This difference is statistically significant, but it is in contrast with literature; this could be explained by the fact that the majority of the implants have been placed in premolars and molars area. ISQ values showed a significant correlation with implants length in maxilla, but no correlations with implants diameter have been found in this study. Those results are partially in contrast with Fuster-Torres et al. [8]: they did not find significative correlation between the implant length and ISQ. The difference in results could be probably explained in view of the limited sample size of that study. No significant differences were also found in ISQ among the different implant types and among the patient characteristics (sex, age, and smoking habits) nor in maxilla nor in mandible. Moreover, in the present study torque showed a significant correlation between implant length and implant diameter both in maxilla and in mandible. Nobel Active implants showed a significantly higher torque than Nobel Replace Select Straight implants in maxilla, Nobel Replace Groovy implants, and Nobel Speedy implants. In mandible Nobel Replace Select Tapered implants had a significantly higher torque than Nobel Replace Select Straight implants. Finally, torque in mandible showed a significant negative correlation with age, unlike other studies [9, 33] which recorded higher torque values in older patients. This fact could be a result of the small difference in age between the patients who have been considered in this study (mean age was 60.31 ± 12.57 years).

## 5. Conclusion

The results of this study suggest that the bone density values (as measured in HU), which were obtained from the CT, could be utilized as predictive parameters during the preoperative assessment when correlated to the primary implant stability (ISQ), especially for the HU values detected for coronal-buccal and middle-lingual. Diameter and implants lengths do not seem to have had correlation to the primary implant stability (ISQ), except for implants lengths in maxilla. Moreover, the results suggest that the cortical thickness, especially in the middle of the ridge, which was measured from the CT, could be utilized as predictive parameter during the preoperative assessment when correlated to the primary implant stability (insertion torque). Subsequently, it is important to take into consideration the Hounsfield Units and the cortical thickness, with regard to the choice of the implant type as well as the surgical technique.

## Figures and Tables

**Table 1:** Patient and intervention characteristics.

Number of patients	75
Mean age ± SD (range)	60.31 ± 12.57 (23–80) years
Females	40 (53.3%)
Smokers	12 (16.0%)
smoking ≤ 10 cigarettes	4 (5.3%)
smoking > 10 cigarettes	8 (10.7%)
# Implants	269
# Patients receiving 1 implant	10 (13.3%)
# Patients receiving 2 implant	22 (29.3%)
# Patients receiving 3 implant	10 (13.3%)
# Patients receiving 4 implant	13 (17.3%)
# Patients receiving 5 implant	5 (6.7%)
# Patients receiving 6 implant	7 (9.3%)
# Patients receiving 7 implant	4 (5.3%)
# Patients receiving 8 implant	1 (1.3%)
# Patients receiving 10 implant	3 (4.0%)
Implant length (mean ± SD)	13.08 ± 1.71 mm
Implant diameter (mean ± SD)	4.36 ± 0.64 mm
Implant type	
Nobel Replace Select Tapered	145 (53.9%)
Nobel Active	40 (14.9%)
Nobel Replace Select Straight	23 (8.6%)
Nobel Replace Groovy	12 (4.5%)
Nobel Speedy	43 (16.0%)
Brånemark Groovy	6 (2.2%)
Dental arch of implant insertion	
Maxilla	149 (55.4%)
Mandible	120 (44.6%)
Implant placement zone	
Anterior (canine - canine)	64 (23.8%)
Posterior (premolars and molars)	205 (76.2%)

SD: standard deviation.

**Table 2:** ISQ comparison among implant types.

	ISQ (*N*; mean ± SD)	*p* value^*∗*^
Maxilla		
Nobel Replace Select Tapered	12; 66.17 ± 7.16	0.201
Nobel Active	19; 63.42 ± 9.53	
Nobel Replace Select Straight	4; 65.50 ± 11.96	
Nobel Replace Groovy	3; 76.00 ± 5.29	
Nobel Speedy	21; 64.81 ± 7.13	
Mandible		
Nobel Replace Select Tapered	30; 72.20 ± 5.94	0.134
Nobel Active	2; 66.00 ± 4.24	
Nobel Replace Select Straight	9; 67.22 ± 4.76	
Nobel Speedy	9; 70.44 ± 7.99	

*N*: number; SD: standard deviation; ^*∗*^one-way ANOVA.

**Table 3:** Maxilla: ISQ comparison among implant types controlling for continuous variables which showed a significant correlation with ISQ (ANCOVA).

	*p* value	*p* value
	(Implant type)	(covariate)
HU1 (coronal-buccal)	0.283	0.040
HU4 (middle-lingual)	0.558	0.157
Implant length	0.059	0.003

**Table 4:** Mandible: ISQ comparison among implant types and implant location.

	Nobel Replace Select Tapered	Nobel Active	Nobel Replace Select Straight	Nobel Speedy	2-way ANOVA
Anterior	64.75 ± 0.96	-	66.25 ± 4.86	67.13 ± 9.90	0.383 (type: 0.396; loc: 0.012)
Posterior	73.35 ± 5.53	66.00 ± 4.24	68.00 ± 5.10	73.10 ± 5.86	
Total	72.20 ± 5.94	66.00 ± 4.24	67.22 ± 4.76	70.44 ± 7.99	

Data are presented as Mean ± Standard deviation; 2-way ANOVA: Two-Way ANOVA with implant type and implant location as independent variables; Two-Way ANOVA results are reported as significance of implant type-implant location interaction (Main effects *p* value).

**Table 5:** Torque comparison among implant types.

	Torque	*p* value^*∗*^
	(*N*; mean ± SD)	
Maxilla		
Nobel Replace Select Tapered	53; 1.68 ± 1.31	<0.001^#§∧^
Nobel Active	38; 2.42 ± 1.03	
Nobel Replace Select Straight	14; 1.21 ± 0.80	
Nobel Replace Groovy	10; 1.00 ± 1.05	
Nobel Speedy	28; 1.54 ± 0.92	
Brånemark Groovy	6; 1.00	
Mandible		
Nobel Replace Select Tapered	92; 2.52 ± 0.99	<0.001^*ç*^
Nobel Active	2; 3.00	
Nobel Replace Select Straight	9; 1.44 ± 0.53	
Nobel Replace Groovy	2; 3.00	
Nobel Speedy	15; 1.93 ± 1.03	

*N*: number; SD: standard deviation; ^*∗*^Kruskal Wallis test. Significant post hoc comparisons: ^#^Nobel Active versus Nobel Replace Select Straight; ^§^Nobel Active versus Nobel Replace Groovy; ^∧^Nobel Active versus Nobel Speedy; ^*ç*^Nobel Replace Select Tapered versus Nobel Replace Select Straight.
